# Study of the Binding Interaction between Wortmannin and Calf Thymus DNA: Multispectroscopic and Molecular Docking Studies

**DOI:** 10.1155/2019/4936351

**Published:** 2019-12-24

**Authors:** Shiva Mehran, Yousef Rasmi, Hamid Reza Karamdel, Ramin Hossinzadeh, Zafar Gholinejad

**Affiliations:** ^1^Department of Biology, Higher Education Institute of Rabe-Rashidi, Tabriz, Iran; ^2^Department of Biochemistry, Faculty of Medicine, Urmia University of Medical Science, Urmia, Iran; ^3^Department of Microbiology, Urmia Branch, Islamic Azad University, Urmia, Iran; ^4^Department of Medical Laboratory Science, Urmia Branch, Islamic Azad University, Urmia, Iran

## Abstract

**Introduction:**

Wortmannin (WTN) is a steroid metabolite that inhibits phosphatidylinositol 3-kinase and other signaling pathways. Structurally, the WTN consists of a cyclopentanophenanthrene-like structure with several oxygen-rich moieties which have the potential to interact with deoxyribonucleic acid (DNA) molecules.

**Methods:**

We aim to evaluate the WTN and calf thymus DNA (ct-DNA) interaction with molecular docking using the AutoDock 4.2 software. UV and fluorescence spectroscopy and viscosity techniques were performed to confirm the *in silico* analysis.

**Results:**

Molecular docking showed that the WTN interacted with ct-DNA via hydrogen bonds at guanine-rich sequences. The number of hydrogen bonds between the WTN and DNA was 1-2 bonds (average 1.2) per WTN molecule. The *in silico* binding constant was 2 × 10^3^ M^−1^. UV spectroscopy showed that the WTN induced a hyperchromic feature without wavelength shifting. The WTN and DNA interaction led to quenching of DNA-emitted fluorescence. The different concentrations of WTN had no effect on DNA viscosity. Taken together, our results demonstrated WTN interacts with DNA in the nonintercalating mode, which is considered as a new mechanism of action.

**Conclusion:**

These results suggest that the WTN may exert its biological effects, at least in part, via interaction with DNA.

## 1. Introduction

**Figure 1:**
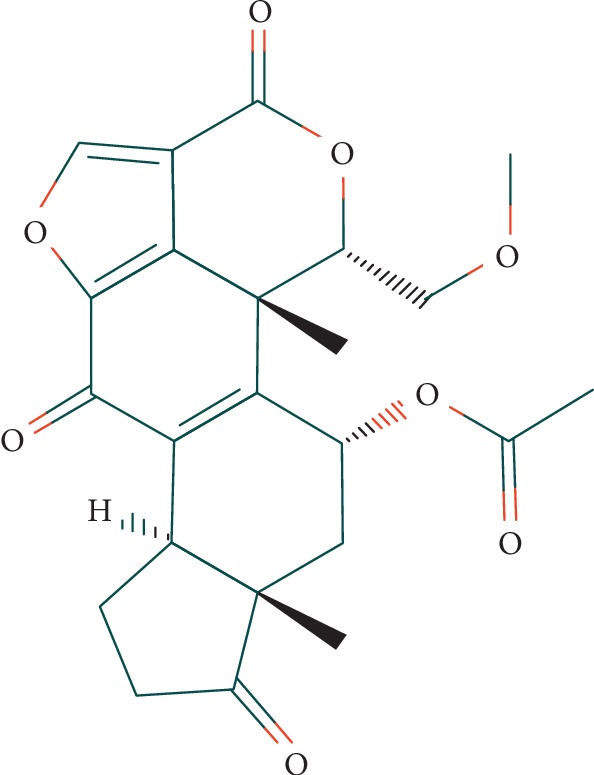
WTN structure (obtained from PubChem [4]).

In 1957, Brian et al. isolated the wortmannin (WTN) from filtrates of *Penicillium wortmannii* Klocker, and Millan et al. proposed the molecular structure based on chemical and spectroscopic evidence, which is illustrated in Figure 1 [1, 2]. This steroid metabolite contains a cyclopentanophenanthrene-like structure, in which A ring is modified to lactone ring and a heterocyclic furan is added to cyclopentanophenanthrene as the fifth ring. WTN belongs to furanosteroids which originate from lanosterol [3].

The subsequent studies confirmed this structure and reported several biologic functions of WTN including antifungal, cell arresting, proapoptotic, and anti-inflammatory activities [5–8]. Due to anti-inflammatory effects of WTN, it may be a therapeutic option to treat of inflammatory disorders such as gout and rheumatoid arthritis.

In 1993, Yano et al. reported that WTN interacts with phosphatidylinositol 3-kinase and inhibits its signaling pathway [9]. The inhibitory effects of WTN on NF-kB and MAPK signaling pathways were also reported [10–12].

Here, we hypothesized that WTN interacts with deoxyribonucleic acid (DNA) molecules that mediate some of its biological function. This hypothesis is based on the evidence indicating that most steroid metabolites modulate the DNA function such as gene expression by direct interaction with DNA and histones [13, 14]. A pioneer study by Cohen and Kidson demonstrated that steroids interact with guanine homoribopolynucleotides predominantly via hydrogen (H) bonds. Polyguanine has maximum hydrogen atoms for H bond formation, and progesterone presents maximum affinity to polynucleotides due to the oxygen-rich structure [15]. On the other hand, WTN contains eight oxygen atoms, which can contribute to the formation of H bonds. In addition, the WTN contains a bay region at the cyclopentanophenanthrene-like structure that may mediate the possible WTN-DNA interaction via the hydrophobic interaction [16].

Therefore, here we propose that WTN may exert its biological effects, at least in part, via interaction with DNA besides the putative inhibitory effects on signaling pathways. Nevertheless, to the best of our knowledge, there is no report regarding *in silico* and/or *in vitro* experiments about the WTN and DNA interaction. In this study, we evaluated WTN and calf thymus DNA (ct-DNA) interaction by molecular docking, viscosity, UV spectroscopy, and fluorescence spectroscopy.

## 2. Materials

Highly polymerized ct-DNA and WTN *Penicillium funiculosum* were purchased from Sigma Chemical Co. (St. Louis, MO, U.S.A.). Tris (hydroxymethyl) aminomethane was obtained from Merck (Darmstadt, Germany). All experiments were performed in Tris buffer solution (10 mM‚ pH 7.4). The stock solution of ct-DNA was prepared by dissolving an appropriate amount of ct-DNA in buffer solution by shaking gently. The stock solution was stored for 24 h at 4°C and utilized not more than 4 days. The concentration of ct-DNA in stock solution was 4.4 × 10^−4^ M that determined by spectrophotometry at 260 nm using an extinction coefficient (*ɛ*) of 6600 M^−1^·cm^−1^. DNA purity was checked using absorption of DNA in 260 nm (A_260_) and 280 nm (A_280_). The A_260_/A_280_ ratio was 1.85, indicating that the ct-DNA was sufficiently pure and protein free [17, 18]. Stock solution of WTN (5 × 10^−3^ M in ddH_2_O) was prepared immediately before experiments due to instability.

## 3. UV Absorption Spectra Analysis

UV spectra analysis is a simple technique for ligand-DNA interaction. Absorbance in 230–300 nm wavelength range was measured, using a spectrophotometer (Analytikjena spekol 2000) equipped with a 1.0 cm quartz cell. For quantification of the WTN-DNA binding constants, the absorption spectra measurements were performed at different concentrations of DNA ((0, 0.66, 1.1, 1.54, 2.2) × 10^−5^ M) and constant WTN concentration (1 × 10^−5^ M).

## 4. Fluorescence Emission Spectra Analysis

Fluorometric assay was performed, using a Shimadzu Spectrophotometer (RF-5301 PC) equipped with a quartz cell of 1.0 cm path length. WTN fluorescence emission spectra were recorded at 280–320 nm by excitation in 260 nm. Analysis was performed at constant concentration of WTN (7.5 × 10^−6^ M) in presence of different ct-DNA concentrations ((0, 0.22‚ 0.66‚1.1, 1.54, 1.98, 2.42‚ 2.86) × 10^−5^ M).

## 5. Viscosity Measurements

The effect of WTN on the viscosity of DNA was measured using a Lovis 2000 M digital micro viscometer. The temperature was kept constant at 25°C. The DNA concentration was 6.6 × 10^−6^ M while varying the WTN concentrations ((0, 0.25, 0.5, 0.75, 1) × 10^−5^ M). The data were presented as (*η*/*η*_0_)1/3 vs [WTN]/[DNA], where *η* and *η*_0_ are the viscosity of ct-DNA in the presence and absence of WTN, respectively [19].

## 6. Molecular Docking Study

Docking study was performed, using the AutoDock Tools 4.2 software. Structure of B-DNA dodecamer (PDB ID: 1BNA) was obtained from the protein data bank (http://www.rcsb.org) [20]. The WTN molecular structure was drawn, and energy was minimized using the HyperChem 8.0.6 software. The DNA file was prepared by deleting water molecules followed by addition of polar H atoms and Gasteiger charge. Grid box dimensions at grid points in *x* × *y* × *z* directions were set to 56‚ 59‚ and 49 with a grid spacing of 0.375 Å. The coordinate centers of grid box were set to *x* = 1.389‚ *y* = 2.417‚ and *z* = 13.472. Lamarckian genetic algorithms were used to carry out molecular docking calculations. The number of runs was set to 100. All other parameters were assigned the default values [21, 22].

## 7. Results and Discussion

### 7.1. UV Absorption Spectroscopy

**Figure 2:**
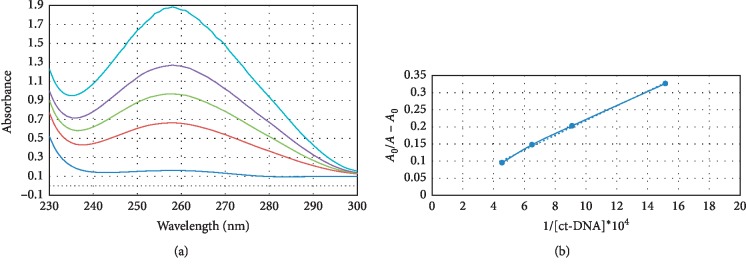
(a) UV spectra of WTN in the presence of different concentrations of ct-DNA (pH 7.4 and room temperature). (b) Plot *A*_0_/*A* − *A*_0_ versus 1/[DNA] for *K*_b_ calculation.

Figure 2(a) shows the UV spectra of the WTN-DNA complex. WTN showed an absorbance at 260 nm, and the peak intensities were enhanced with increasing the concentration of ct-DNA. A hyperchromic effect without red or blue shift was observed for all molar ratios, which demonstrated there is a nonintercalative binding interaction [23, 24]. The observed spectral changes proposed that WTN interacts with DNA at the groove-binding model [23]. The binding constant (*K*_b_) was calculated using equation (1) from UV data [25].

(1) $$\begin{array}{c} \frac{A_{0}}{A-A_{0}}=\frac{\varepsilon_{G}}{\varepsilon_{H-G}-\varepsilon_{G}}+\frac{\varepsilon_{G}}{\varepsilon_{H-G}-\varepsilon_{G}}\times \frac{1}{K_{\text{b}}\left[\text{DNA}\right]}, \end{array}$$

where *A*_0_ and *A* are the absorbance of WTN in the absence and presence of ct-DNA, respectively. The *ɛ*_*G*_ and *ɛ*_*H*−*G*_ are the absorption coefficients of WTN and WTN-DNA complex, respectively. The *K*_b_ is 2 × 10^3^ M^−1^ obtained by plotting *A*_0_/*A* − *A*_0_ versus 1/[DNA] (Figure 2(b)). This value for *K*_b_ suggested the groove binding model that is similar to previous studied agents (e.g., Cu–Sn_2_ complex, 1.67 × 10^4^ M^−1^ [26], metformin complex, 8.3 × 10^4^ M^−1^ [27], Ho(phen)^2^Cl^3^_H_2_O (1.36 × 10^4^ M^−1^ [28], adefovir dipivoxil, 3.33 ± 0.2 × 10^4^ M^−1^ [20], and sorafenib complex, 5.6 × 10^3^ M^−1^ [29]).

### 7.2. Fluorescence Spectra

**Figure 3:**
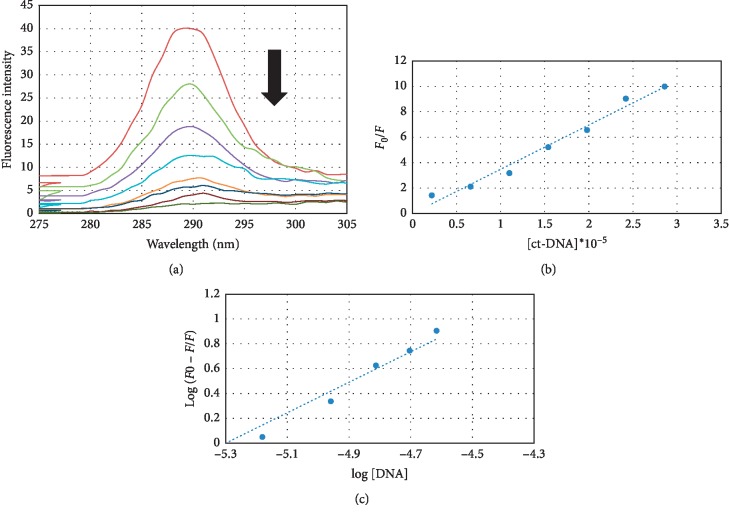
(a) Fluorescence emission spectra of WTN in the presence of different concentrations of ct-DNA. (b) Stern–Volmer plot of *F*_0_/*F* versus [*Q*]. (c) Plot of log [(*F*_0_ − *F*)/*F*] versus log [DNA].

The fluorescence emission spectra of WTN alone and in the presence of ct-DNA are shown in Figure 3(a). WTN has emission spectra with maximum emission at about 291 nm when excited at 260 nm. The fluorescence of WTN was quenched by ct-DNA without any shift in maximum emission in a concentration-dependent manner. Reduced emission intensities (quenching) confirmed WTN-DNA interaction. Fluorescence quenching is described by the Stern–Volmer equation (equation (2)) [30, 31]:

(2) $$\begin{array}{c} \frac{F_{0}}{F}=1+K_{\text{q}}\tau_{0}\left[Q\right]=1+\;K_{\text{sv}}\left[Q\right], \end{array}$$

where *F*_0_ and *F* denote the steady state fluorescence intensities in the absence and presence of the quencher *Q*, respectively, *K*_q_ is the quenching rate constant of the biomolecule, *τ*_0_ is the average lifetime of the molecule without the quencher, [*Q*] is the quencher concentration, and *K*_sv_ is the Stern–Volmer quenching constant.  Figure 3(b) shows the Stern–Volmer plots of *F*_0_/*F* versus [*Q*].

The calculated *K*_sv_ value was 3.5 × 10^5^, which is higher than the classical groove binding small molecule [32–34]. This severe quenching phenomenon indicated that WTN interacts with DNA strongly and probably via the intercalation model.

Apparent binding constant (*K*_b_) and the binding stoichiometry (*n*) were calculated according to Zhang et al.'s equation (3) [35]:

(3) $$\begin{array}{c} \log\left[\frac{\left(F_{0}-F\right)}{F}\right]=\log\;K_{\text{b}}+n\;\log\left[Q\right], \end{array}$$

where *F*_0_ and *F* are the fluorescence intensity in the absence and presence of a quencher at various concentrations [*Q*], respectively.  Figure 3(c) shows the plot of log [(*F*_0_ − *F*)/*F*] versus log [DNA]. The intercept is about 1.47, which indicates WTN binds to ct-DNA in more than one position. This observation is consistent with *in silico* docking analysis, which indicates in some positions WTN binds to ct-DNA via two H bonds.

### 7.3. Viscosity Study

**Figure 4:**
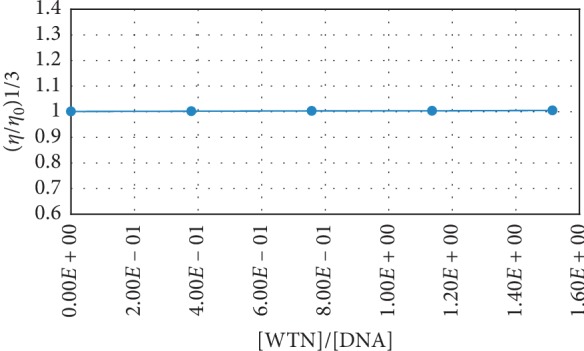
Viscosity of ct-DNA in constant concentration (6.6 × 10^−6^) at 25°C in presence of WTN at different concentrations ((0, 2.5, 5, 7.5, 10) × 10^−6^).

Viscosity experiment is an effective tool to determine the binding mode between small molecules and ct-DNA [36]. In the intercalative binding mode, the DNA double helix separation and conformational change result in an increase in DNA viscosity while groove binding interaction has no significant effect on the DNA viscosity [37, 38]. The effect of WTN on the viscosity of ct-DNA at 25°C is shown in Figure 4. There is no significant change in the ct-DNA viscosity by increasing concentration of WTN. This observation suggests that the groove binding is the mode of interaction between WTN and ct-DNA.

### 7.4. Molecular Docking Study

**Figure 5:**
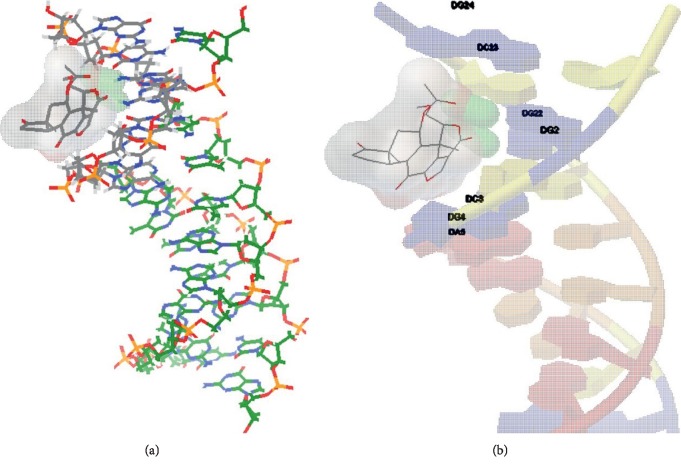
(a) *In silico* groove binding of WTN and DNA. (b) H bond (green) between WTN and guanine.

Molecular docking study is an appealing method to understand the interaction between ligands and DNA [39]. Molecular models were made to discuss the binding modes using the AutoDock software for the interactions of WTN with ct-DNA (PDB ID: 1BNA). The structure of WTN was drawn and subjected to energy optimization [40].100 docking runs were successfully carried out‚ and the obtained runs data for the conformers are listed in Table 1. The first conformation was taken from one of the lowest binding energy docking conformation. Our results showed WTN incorporated to DNA and the H bond formed between amino groups at C-2 on guanine and oxygen atom of the acetoxy group on C-11. In some conformations, the oxygen atom of furan and lactone rings was involved in H bond formation. There are 63 groups in which H bonds formed, including 39 groups with single H bond with guanine, 12 groups with two H bonds simultaneously with guanine and cytosine‚ 11 groups with cytosine, and 1 group with two H bonds between one WNT and two guanines. These results proposed that WTN have interaction predominantly with guanine. The energetically most desirable conformation of the docked pose is shown in Figure 5.

## 8. Conclusion

The results of this study indicated that WTN interacts with DNA molecules. Our results did not provide strong evidence about the mode of interaction but based on *in silico* molecular docking, UV absorption spectroscopy, fluorescence emission spectroscopy, and viscosity measurement, a fluctuation between intercalation and groove binding model. The H bond may be an important force in this interaction where DNA is a hydrogen donor and the WTN oxygen is considered as a H acceptor. To the best of our knowledge, this is the first report that revealed a new and nontraditional mechanism for the biologic effects of WTN.

Taken together lack of a red-blue shift in UV spectra and the value of obtained *K*_b_, it is suggested the groove-binding mode. The UV spectra results and groove-binding mode were confirmed by *in silico* analysis. However, the high *K*_sv_ value suggests the intercalation mode of interaction.

As a third mechanism, it is possible that the interaction may occur through both groove binding and intercalation, which was reported for some molecules previously [41, 42].

Circular dichroism, isothermal titration calorimetry, and ionic strength and melting temperature experiments could be performed to elucidate the precise interaction mode. However, they were not carried out due to WNT instability. In addition, it should be determined whether the WTN-DNA interaction influences the gene expression and other biologic functions. Taken together, these results suggest that the WTN may exert its biological effects, at least partly, by interaction with DNA besides the putative signaling pathway inhibitory properties.

## Figures and Tables

**Table 1:** The data on DNA and WTN docking obtained from the AutoDock software.

Rank	Run	Lowest binding energy (kcalM^−1^)	Mean binding energy (kcalM^−1^)	Number of cluster	Ki (*μ*M)	Cluster RMSD	Reference RMSD
1	8	−6.83	−6.61	28	9.84	0.00	30.84
2	63	−6.28	−6.23	29	24.88	0.00	32.52
3	37	−6.20	−6.08	13	28.35	0.00	31.86
4	31	−6.19	−6.04	4	29.09	0.00	29.51
5	23	−6.11	−6.01	12	33.43	0.00	31.40
6	4	−5.99	−5.96	3	40.75	0.00	34.94
7	80	−5.91	−5.86	11	46.82	0.00	31.71

## Data Availability

The data used to support the findings of this study are available from the corresponding author upon request.
